# Fractalkine Modulates Microglia Metabolism in Brain Ischemia

**DOI:** 10.3389/fncel.2019.00414

**Published:** 2019-09-13

**Authors:** Clotilde Lauro, Giuseppina Chece, Lucia Monaco, Fabrizio Antonangeli, Giovanna Peruzzi, Serena Rinaldo, Alessio Paone, Francesca Cutruzzolà, Cristina Limatola

**Affiliations:** ^1^Department of Physiology and Pharmacology, Sapienza University of Rome, Rome, Italy; ^2^Department of Molecular Medicine, Laboratory Affiliated to Istituto Pasteur Italia – Fondazione Cenci Bolognetti, Sapienza University of Rome, Rome, Italy; ^3^Center for Life Nano Science@Sapienza, Istituto Italiano di Tecnologia, Rome, Italy; ^4^Department of Biochemical Sciences “A. Rossi Fanelli”, Sapienza University of Rome, Rome, Italy; ^5^Department of Physiology and Pharmacology, Laboratory Affiliated to Istituto Pasteur Italia – Fondazione Cenci Bolognetti, Sapienza University of Rome, Rome, Italy; ^6^IRCCS NeuroMed, Pozzilli, Italy

**Keywords:** CX3CL1, microglia, ischemia, neuroprotection, neuroinflammation

## Abstract

In the CNS, the chemokine CX3CL1 (fractalkine) is expressed on neurons while its specific receptor CX3CR1 is expressed on microglia and macrophages. Microglia play an important role in health and disease through CX3CL1/CX3CR1 signaling, and in many neurodegenerative disorders, microglia dysregulation has been associated with neuro-inflammation. We have previously shown that CX3CL1 has neuroprotective effects against cerebral ischemia injury. Here, we investigated the involvement of CX3CL1 in the modulation of microglia phenotype and the underlying neuroprotective effect on ischemia injury. The expression profiles of anti- and pro-inflammatory genes showed that CX3CL1 markedly inhibited microglial activation both *in vitro* and *in vivo* after permanent middle cerebral artery occlusion (pMCAO), accompanied by an increase in the expression of anti-inflammatory genes. Moreover, CX3CL1 induces a metabolic switch in microglial cells with an increase in the expression of genes related to the oxidative pathway and a reduction in those related to the glycolytic pathway, which is the metabolic state associated to the pro-inflammatory phenotype for energy production. The data reported in this paper suggest that CX3CL1 protects against cerebral ischemia modulating the activation state of microglia and its metabolism in order to restrain inflammation and organize a neuroprotective response against the ischemic insult.

## Introduction

### CX3CL1 and CX3CR1 in CNS

The chemokine CX3CL1, also known as fractalkine, and its receptor CX3CR1 are expressed by immune and non-immune cells throughout organisms and their expression is cell type-unique in each tissue. In the brain, CX3CL1 is mostly expressed by neurons (Harrison et al., 1998) and its expression is reported on astrocytes upon inflammatory stimulations (Yoshida et al., 2001; Guillemin et al., 2003) while CX3CR1 is expressed on parenchymal microglia (Harrison et al., 1998) and in perivascular, subdural meningeal and choroid plexus macrophages, which, together with microglia, are critical regulators of immune responses in CNS upon neuroinflammation (Jung et al., 2000; Prinz et al., 2011; Goldmann et al., 2013, 2016; Yona et al., 2013). CX3CR1 signaling involves phospholipase C (PLC), phosphatidylinositol 3-kinases (PI3K) and extracellular signal–regulated kinases (ERKs), and the recruitment of transcription factors such as nuclear factor kappa-light-chain-enhancer of activated B cells (NF-κB) and cyclic adenosine monophosphate response element binding protein (CREB) (Sheridan and Murphy, 2013).

### Metabolic Reprograming in the Regulation of the Innate Inflammatory Response

Microglia, under physiological conditions, continuously monitor the surrounding parenchyma by extending and retracting cellular processes to detect alteration of brain homeostasis (Davalos et al., 2005; Nimmerjahn et al., 2005) such as those induced by inflammatory stimuli. Increasing evidence suggest a role of metabolic reprograming in the modulation of the innate inflammatory response (Orihuela et al., 2016). For instance, it is known that polarization toward a pro-inflammatory phenotype induces peripheral macrophages to change from the oxidative phosphorylation to anaerobic glycolysis, so as to increase adenosine triphosphate (ATP) production (Kelly and O’Neill, 2015). Modification of metabolic functions from the ability to promote cell proliferation like anti-inflammatory phenotype to a killing/inhibitory capacity in the pro-inflammatory state, allows macrophages to respond with proper functions in different context (Mills et al., 2000; Rodríguez-Prados et al., 2010; Odegaard and Chawla, 2011; Biswas and Mantovani, 2012). A similar metabolic switch has been well characterized in tumor cells: it is known as the Warburg effect (Warburg, 1956) and permits tumor cells to maintain their increased energy demand (Griffin and Shockcor, 2004). Also microglial cells are capable of an adaptable use of energy substrates: similar to macrophages, when stimulated, they switch among oxidative phosphorylation and glycolytic metabolism. In particular, microglial activation with lipopolysaccharide (LPS) augmented lactate production, diminished mitochondrial oxygen consumption and mitochondrial ATP production, resulting in the increase of glycolysis and the decrease of oxidative phosphorylation (Voloboueva et al., 2013). Moreover, primary rat microglia cultured with increasing glucose concentration (from 10 mM to 50 mM) boosted tumor necrosis factor α (TNFα) secretion (Quan et al., 2011; Zhang et al., 2015). On the other hand, IL-4-stimulated BV-2 cells decreased glucose consumption and lactate production (Gimeno-Bayón et al., 2014) and primary murine microglia stimulated with IL-4/IL-13 maintained an oxidative metabolic state (Orihuela et al., 2016) suggesting that this shift was associated with a reduced need for anabolic reactions. Since neuro-inflammation caused by microglia hyperactivity has been associated with several neurodegenerative diseases (Cartier et al., 2014) and many evidence exist of a metabolic reprograming of microglia in neurodegeneration (Ulland et al., 2017), a metabolic switch toward oxidative metabolism might contribute to afford the healthful role of microglia in some pathophysiological conditions, resulting in the production of metabolites which are beneficial for neurons.

### Neuroprotective Activity of CX3CL1 During Ischemia

It was reported that CX3CL1/CX3CR1 signaling in microglia plays important roles in physiological and pathological conditions (Lauro et al., 2015). Many evidence suggest that CX3CL1 has dual activities in the CNS, with either beneficial or detrimental potentials, depending on the activation state of microglia (Lauro et al., 2015). Furthermore, the administration of exogenous CX3CL1 reduces ischemic damage *in vivo* (Cipriani et al., 2011). Ischemia is the second leading cause of death in human and can lead to permanent disability (Johnson et al., 2016). It occurs when cerebral artery blood flow is reduced by a thrombus or atherosclerotic plaque, causing an abrupt deprivation of oxygen and nutrients into the brain. Currently the treatment for cerebral ischemia is reperfusion by thrombolytic administration or surgery, in order to reduce the volume of acute ischemia and improve the clinical outcome. However, only 10–20% of stroke patients receive a prompt therapy because the time window to restore blood flow to a cerebral artery is approximately 4 h from first symptoms and the risk of cerebral hemorrhage is high after this time (Chaudhary et al., 2017). Animal models of cerebral ischemia describe a well-established timing of inflammatory events after brain injury: in particular, it was demonstrated that microglia phenotype changes from anti- to pro-inflammatory with the progression of cerebral ischemia (Fumagalli et al., 2015; Ma et al., 2017). Initially, few minutes after the onset of ischemia, resident microglial cells acquire an anti-inflammatory phenotype, mainly in the peri-infarct region, to constrain brain damage. At 6 days upon ischemic insults, pro-inflammatory microglia predominate in the region close to the infarct zone (Schroeter et al., 1997; Perego et al., 2011). This microglia release reactive oxygen species and pro-inflammatory cytokines that prompt the activation of cerebrovascular endothelial cells and support the adhesion and transmigration of leukocytes into the injured tissue, contributing to the spread of brain damage (Kriz, 2006; Ceulemans et al., 2010; Jin et al., 2010; Grønberg et al., 2013). The inflammatory infiltrate induces anoxic depolarization, perturbs glutamatergic neurotransmission and increases the levels of intracellular calcium, causing the formation of reactive oxygen species and neuronal death (Ceulemans et al., 2010). However, inflammatory cells might also have a protective effects: resident microglia/macrophages achieve phagocytosis and produce neurotropic factors such as neurotrophins and tumor growth factor β1 (TGF β1), both involved in neuroprotection and tissue repair (Jin et al., 2010). Animal models of cerebral ischemia demonstrate that increased pro-inflammatory polarization of microglia is associated with a larger infarct area whereas anti-inflammatory microglia resolve inflammation, limit stroke injury progression and promote tissue reparation (Iadecola and Anrather, 2011). This experimental evidence suggests that a targeted modulation of microglia could be used to reduce the extent of tissue damage. Our previous study showed that CX3CL1 has neuroprotective effect against cerebral ischemia. Here, we investigated the involvement of CX3CL1 in microglia phenotype and metabolic switch toward oxidative metabolism and the underlying neuroprotective effect toward ischemia injury. The expression profiles of anti- and pro-inflammatory genes and those related to the metabolic reprograming following the inflammatory response were detected *in vitro* after CX3CL1 stimulation of microglial primary cultures and *in vivo* after permanent middle cerebral artery occlusion (pMCAO) in mice, in the presence of CX3CL1, to confirm the *in vitro* data and also to verify a possible role of CX3CL1 in modulating microglia polarization state upon ischemia development. In this paper we demonstrated that CX3CL1 inhibits microglial pro-inflammatory phenotype and induces an increase in the expression of anti-inflammatory genes. Moreover, it induces a metabolic switch with an increased expression of genes related to the oxidative pathway and a reduction in those related to glycolytic one, which is the metabolic state associated to the pro-inflammatory phenotype for energy production, suggesting that CX3CL1 protects against cerebral ischemia injury modulating the activation state of microglia and its metabolism in order to restrain inflammation and activate a neuroprotective response against the ischemic insult.

## Materials and Methods

### Materials

Recombinant human CX3CL1 (cat#300-31) was from Peprotech; IL-4 (cat#12340045) was from Immunotools; LPS (cat#L4391) was from Sigma-Aldrich; anti-Arg1 antibody was from Santa Cruz (cat#sc-271430 RRID:AB_10648473); anti-Actin antibody (cat#A2066) was from Sigma-Aldrich. Secondary antibodies were from DAKO; Microbeads CD11b+ were from Miltenyi Biotec. All cell culture media, fetal bovine serum (FBS), goat serum, penicillin G, streptomycin, glutamine and Hoechst (cat#33342, RRID:AB_10626776) were from Invitrogen; poly-L-lysine (cat#P2636) and papain were from Sigma-Aldrich. Griess reagent kit for Nitrite determination was from Molecular Probe (cat#G-7921), Lactate Assay Kit (cat#MAK064) and Arginase Activity Assay Kit (cat#MAK112) were from Sigma-Aldrich; Seahorse Cell Mito Stress Test Kit for Seahorse XFe Analyzer Respiratory Assay reagents were from Agilent (cat#103015-100).

### Animals and Cell Cultures

The experiments described in the present work, were approved by the Italian Ministry of Health in accordance with the guidelines on the ethical use of animals from the European Community Council Directive of September 22, 2010 (2010/63/EU). Wild type mice C57BL/6J (cat# JAX: 000664, RRID: IMSR_JAX:000664) were from Jackson Laboratory.

### Microglia Culture and Polarization

Microglial cells were obtained from mixed glia cultures derived from the cerebral cortices of post-natal day 0–2 (p0–p2) *wt* mice. Cortices were chopped and digested in 15 U/ml papain for 20 min at 37°C. Cell suspensions were plated (5 × 10^5^ cells/cm^2^) on poly-L-lysine (0.1 mg/ml) coated flasks in growth medium supplemented with 10% FBS. After 9–11 days, cultures were shaken for 2 h at 37°C to detach and collect microglia cells. These procedures gave almost pure microglial cell populations as previously described (Lauro et al., 2010). For microglia polarization, cells were seeded on poly-L-lysine coated 12 well plates (40 × 10^4^ cells), 24-well plate (20 × 10^4^ cells) or 12 mm glass (80 × 10^3^ cells) slides and 2 days after they were treated with LPS 100 ng/ml or IL-4 20 ng/ml for 24 h and with CX3CL1 100 nM for further 24 h.

### Form Factor Calculation

Microglia were seeded on 12 mm glass coverslips (80 × 10^3^ cells), treated as reported, fixed, permeabilized, blocked and stained with Alexa-Fluor 488 Phalloidin (Invitrogen) for 20 min together with Hoechst. For the form factor calculation we used 3 different primary microglial culture preparations, 2 glass coverslips for each different conditions and we counted 20 cells for each glass coverslips randomly selected. Fluorescent images were processed using the MetaMorph 7.6.5.0 software (Molecular Device, Sunnyvale, CA, United States), and form factor was calculated according the formula: 4π area/perimeter^2^ (Neubrand et al., 2014). Form factor is a parameter taken as 1 for round cells, and correspondingly <1 when the morphology deviates from the spherical shape.

### Permanent Middle Cerebral Artery Occlusion (pMCAO)

Mice (25–28 g, 11–12 weeks) were anesthetized with chloral hydrate (400 mg/kg, i.p.). The right middle cerebral artery (MCA) was permanently occluded by electrocoagulation as described previously (Storini et al., 2006). Mice were maintained at 37°C during surgery and sacrificed 24 or 72 h after pMCAO: they were deeply anesthetized and intracardially perfused with ice cold PBS.

### Drugs and Administration Protocols

CX3CL1 was dissolved in saline and intra-cerebro-ventricular injected 20 min before pMCAO at 70 pmol/2 μl in mice similar to what was used previously (Cipriani et al., 2011). Anesthetized animals were immobilized on a stereotaxic apparatus (David Kopf Instruments) and injected in the right cerebral ventricle [1 mm lateral and 3 mm deep, according to the atlas of Paxinos and Watson (1998)]. A constant rate of infusion (0.2 μl/min) was maintained with a pump (KD Scientific). Control-operated animals received only vehicles.

### Isolation of CD11b^+^ Cells

Mice 24 and 72 h after ischemia were deeply anesthetized and intracardially perfused with ice cold PBS. Brains were removed, each hemisphere was cut into small pieces and disrupted in a glass-teflon homogenizer. Cell suspension was first applied to a 30-μm cell strainer and then labeled with CD11b^+^ Microbeads, loaded onto a MACS Column (Miltenyi Biotec) and placed in the magnetic field of a MACS Separator. After removing the magnetic field, CD11b^+^ cells were eluted and used for RNA extraction. With this procedure we usually obtain around 80-50 × 10^3^ cells from one hemisphere, the viability is about 70% and the purity 99%.

### Isolation of Microglia Cells by Fluorescence-Activated Cell Sorting (FACS)

Cell suspensions were obtained as above and passed through a 100 μm nylon cell strainer (Becton Dickinson). The suspension was centrifuged (800 *g*, 10 min, RT), the pellet resuspended in 4 ml of 30% Percoll (Sigma) and overlaid on the top of HBSS. The suspension was centrifuged (14000 *g*, 15 min, RT), the pellet was resuspended in 2% BSA in PBS without Ca^2+^ Mg^2+^. Single cell suspension was washed in staining buffer (PBS without Ca^2+^ Mg^2+^, 0.5% BSA, 2 mM EDTA, 0.025% NaN_3_). Anti-CD16/32 (clone 24G2) was added (10 min) to prevent non-specific and Fc-mediated binding. Then, cells were stained with the following indicated antibodies for 20 min at 4°C. Directly conjugated mAbs for the following antigens (clone name) were used: CD45.2 APC-eFluor 780 (104) from eBioscience, CD11b PE-Cy7 (M1/70), Ly6C APC (HK1.4), Ly6G PE (1A8) from BioLegend. Cells were sorted using a FACSAriaIII (BD Biosciences) equipped with lasers at 561 and 633 nm, and FACSDiva software (BD Biosciences version 6.1.3). Data were analyzed using FlowJo software (Tree Star, version 9.3.2). Briefly, cells were first gated based on morphology using forward versus side scatter parameters (FSC-A versus SSC-A) and then doublets were excluded considering morphology parameter area versus width (A versus W). Starting from CD45 positive low population, microglia cells were isolated as CD11b positive Ly6C/Ly6G double negative cells. Cells were collected in 1.5 mL eppendorf tubes for later RNA extraction. Following isolation, an aliquot of each tube with sorted cells was evaluated for purity at the same instrument resulting in an enrichment >99% for each sample. With this procedure we obtain around 20 × 10^3^ cells from one hemisphere.

### Quantitative Real-Time PCR (RT-qPCR)

Samples were lysed in Trizol reagent (Invitrogen) for isolation of total RNA. The quality and yield of RNAs were verified using NANODROP One (Thermo Fisher Scientific). For RT-qPCR, Reverse transcription reaction was performed in a thermocycler using IScript TM RT Supermix (Biorad) under the following conditions: incubation, 25°C, 5′; reverse transcription, 42°C, 45′; inactivation, 85°C, 5′. Real Time-PCR was carried out in a I-Cycler IQ Multicolor RT-PCR Detection System using SSO Fast Eva Green Supermix (Biorad). The PCR protocol consisted of 40 cycles at 95°C, 30″ and 60°C, 30″. For quantification analysis, the comparative Threshold Cycle (Ct) method was used. The Ct values from each gene were normalized to the Ct value of GAPDH in the same cDNA samples. Relative quantification was performed using the 2^–ΔΔ*Ct*^ method (Schmittgen and Livak, 2008) and expressed as fold increase in arbitrary values. Primers sequences are reported in Table 1.

**TABLE 1:** Sequences of the primers used for RT-qPCR.

**Gene**	**Species**	**Primer forward**	**Primer reverse**
*inos* *il1β* *cd86* *tnfα* *arg1* *ym1* *fizz1* *gapdh* *il6* *pgc1a* *pparg* *tgfb1* *tgfb1r* *prc* *sirt3* *g6pdh* *aldoA* *eno1* *hk1* *ldha* *pklr* *slc25a15* *pkm2*	Mouse Mouse Mouse Mouse Mouse Mouse Mouse Mouse Mouse Mouse Mouse Mouse Mouse Mouse Mouse Mouse Mouse Mouse Mouse Mouse Mouse Mouse Mouse	ACATCGACCCGTCCACAGTAT GCAACTGTTCCTGAACTCAACT AGAACTTACGGAAGCACCCA GTGGAACTGGCAGAAGAG CTCCAAGCCAAAGTCCTTAGAG CAGGTCTGGCAATTCTTCTGAA CCAATCCAGCTAACTATCCCTCC TCGCTCCCGTAGACAAAATGG GATGGATGCTACCAAACTGGA ACAGCTTTCTGGGTGGATTG TCCGTGATGGAAGACCACTCGCA GGAGAGCCCTGGATACCAAC GCTCCTCATCGTGTTGGTG GCTTGGCTGTAGGAAACTCAG ACAGCTACATGCACGGTCTG TTATCATCATGGGTGCATCG TTAGTCCTTTCGCCTACCCA CACCCTCTTTCCTTGCTTTG ACCAAAAATACGACCCCCTC CCGTTACCTGATGGGAGAGA GAACACCTCTGCCTTCTGGA AGTGGTTGGATTGGATCAGC CAGCAGGAACCGAAGTACG	CAGAGGGGTAGGCTTGTCTC ACTTTTTGGGGTCCGTCAACT GGCAGATATGCAGTCCCATT CCATAGAACTGATGAGAGG AGGAGCTGTCATTAGGGACATC GTCTTGCTCATGTGTGTAAGTGA ACCCAGTAGCAGTCATCCCA TTGAGGTCAATGAAGGGGTC TCTGAAGGACTCTGGCTTTG TGTCTCTGTGAGGACCGCTA TCAGCAACCATTGGGTCAGCTCT AAGTTGGCATGGTAGCCCTT CAGTGACTGAGACAAAGCAAAGA CAGTTCTGGGGCTTGTAACC TGCTCCCCAAAGAACACAAT GTCGTCCACTGTGAGTCGTG GCGATGTCAGACAGCTCCTT AGATCGACCTCAACAGTGGG GGGTTATAATCCCGGGAGAG TGCCCAGTTCTGGGTTAAGA AATGTTCATCCCTGCCTTGA CATTTCACAAGCTCCGTGG TGTGTTCCAGGAAGGTGTCA

### Nitric Oxide (NO) Measurement

Nitric Oxide production by microglia cultures was assessed by measuring nitrite accumulation in the culture medium by Griess Reagent Kit according to manufacturer’s instructions (Molecular Probes, MA, United States). For the NO measurements we used five different primary microglial culture preparations. The absorbance was measured at 570 nm in a spectrophotometer microplate reader (BioTek Instruments Inc., Winooski, VT, United States).

### Arginase Activity Assay Measurement

Arginase activity in microglia cultures was assessed by measuring urea accumulation in the culture medium by Ariginase activity assay Kit according to manufacturer’s instructions (Sigma-Aldrich). For the Ariginase activity measurements we used six different primary microglial culture preparations. The absorbance was measured at 430 nm in a spectrophotometer microplate reader (BioTek Instruments Inc., VT, United States).

### Lactate Measurement

Lactate production by microglia cultures was assessed by measuring lactate accumulation in the culture medium by Lactate assay Kit according to manufacturer’s instructions (Sigma-Aldrich). For the lactate measurements we used three different primary microglial culture preparations. The absorbance was measured at 570 nm in a spectrophotometer microplate reader (BioTek Instruments Inc., Winooski, VT, United States).

### Seahorse XF Analyzer Respiratory Assay

Cellular oxygen consumption rate (OCR) and extracellular acidification rate (ECAR) were detected using XF Cell Mito Stress Test (Agilent) measured by the extracellular flux analyzer XFe96 (Seahorse Bioscience, Houston, TX, United States) in the Hyp-ACB Platform in Sapienza University. Microglia were cultured on XFe culture miniplates (coated with poly-L-Lys) for a total of 4 days (40 × 10^3^ cells/well). After 2 days microglia were stimulated with 100 or 10 ng/ml of LPS for 24 h and 100 nM fractalkine for further 24 h. Both treatments show the same trend but only the change observed with 10 ng/ml was statistically significant and therefore only these data were shown. It is likely that 100 ng/ml treatment leads to a dramatic OCR reduction and in such a contest the variation due to CX3CL1 treatment are below the sensibility of the system. The sensor cartridge for XFe analyzer was hydrated in a 37°C non-CO_2_ incubator a day before the experiment. According to the manufacturer instructions, stressors concentrations were optimized and added as follows: 1 μM oligomycin as complex V inhibitor, 1 μM FCCP (uncoupler agent) and 0.5 μM rotenone/antimycin A (inhibitors of complex I and III). During sensor calibration, cells were incubated 1 h in a 37°C non-CO_2_ incubator in 180 μl assay medium (XF base medium supplemented with 10 mM glucose, 10 mM pyruvate and 2 mM L-glutamine at pH 7.4, was used to wash the cells and replace the growth medium). OCR was normalized for total protein/well/40 × 10^3^ cells. Each sample/treatment was analyzed in at least 8 wells for experiment; the figure represents one sample experiment. Two independent experiments were carried out on two different primary microglial culture preparations.

### Western Blotting Analysis

For protein analysis, microglial cells were seeded on 12 well plates (40 × 10^4^ cells) and treated with LPS (100 ng/ml) for 24 h and the day after with CX3CL1 (100 nM) for further 24 h; cells were washed with PBS and lysed in hot 2× Laemmli buffer, boiled 5 min and sonicated. The same amount of protein samples was separated on 12% SDS-polyacrylamide gel electrophoresis and analyzed by western immunoblot using the following primary antibodies: Arg-1 (1:200, Santa Cruz Biotechnology) PKM2 (1:2000, Cell Signaling), Actin (1:2000 Sigma-Aldrich), HRP-tagged goat anti-mouse and anti-rabbit IgG were used as a secondary antibody (1:2000; Dako). For protein analysis we used four different primary microglial culture preparations. Detection was performed through the chemiluminescent assay Immun-Star Western C Kit (Bio-Rad, CA) and densitometric analysis was carried out with Quantity One software (Bio-Rad, CA).

### Statistical Analysis

Data are expressed as the means ± SEM. Student’s *t*-test, paired *t*-test, one-way analysis of variance (ANOVA) was performed. A value of *P* < 0.05 was considered significant. All statistical analyses were carried out using the Sigma Plot 11.0 Software (Systat Software GmbH, Erkrath, Germany).

## Results

### CX3CL1 Induces Change in the Polarization State of Microglia in Culture

**FIGURE 1:**
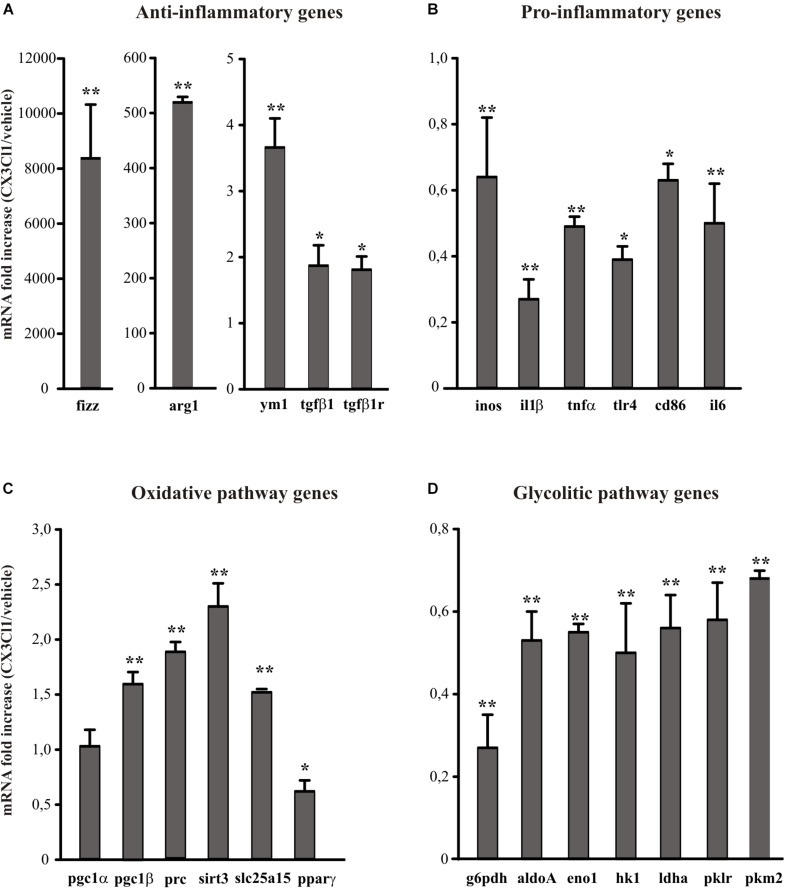
Effects of CX3CL1 in modulating microglia polarization state. Expression analysis by RT-qPCR for mRNAs of anti-inflammatory (**A**: *arg1, ym1*, *fizz, tgfβ1, tgfβ1r*), pro-inflammatory (**B**: i*nos, il1β, tnfα, tlr4, cd86, il6*) oxidative pathway (**C**: *pgc1*α, *pgc1*β, *prc*, *sirt3*, *slc25a15 ppar*γ) and glycolytic pathway (**D**: *g6pdh, aldoA, eno1, hk1, ldhα, pklr, pkm2*) related genes in primary *wt* microglia treated with CX3CL1 (100 nm, 24 h). For each gene data are expressed as specific mRNA fold increase in CX3CL1 treated cells normalized to specific mRNA expression in vehicle. Statistical analysis: Data are expressed as the mean (±SEM.) of *n* = 8, ^∗∗^*p* < 0.001, ^∗^*p* < 0.05, Student’s *t-*test.

Since CX3CL1 is neuroprotective in ischemia (Cipriani et al., 2011) and it is known that neuro-inflammation plays a role in brain damage following ischemic insult (Iadecola and Alexander, 2001; Cheon et al., 2017), we wanted to verify the hypothesis that the neuroprotective effect of CX3CL1 was due to its ability to modulate the phenotype of microglia. For this reason, we used primary murine microglial cells, treated them with CX3CL1 (100 nM) for 24 h and analyzed the expression levels of different anti- (*ym1, arg1, fizz, tgfβ1, tgdβ1r*) and pro- (*inos*, il1β, tnfα, tlr4, cd86, il6) inflammatory genes (Michelucci et al., 2009; Gabrusiewicz et al., 2011) by quantitative real time PCR (RT-qPCR). Data reported in Figure 1 show that cells exposure to CX3CL1 increased the expression of anti-inflammatory (Figure 1A, *n* = 8 ^∗∗^*p* < 0.001, ^∗^*p* < 0.05; Student’s *t*-test) and lowered the expression of pro-inflammatory genes (Figure 1B, *n* = 8 ^∗∗^*p* < 0.001, ^∗^*p* < 0.05; Student’s *t*-test). Since numerous evidence suggest that upon different activation stages microglial cells switch from the oxidative phosphorylation to the anaerobic glycolysis in order to increase ATP production (Voloboueva et al., 2013; Gimeno-Bayón et al., 2014) we decided to investigate whether CX3CL1 treatment could modify the metabolic repertoire of microglia. Data obtained indicate that CX3CL1 increased the transcription of some genes involved in the oxidative pathway (*pgc1*α, *pgc1*β, *prc*, *sirt3*, *slc25a15*, *ppar*γ) (Figure 1C, *n* = 8 ^∗∗^*p* < 0.001, ^∗^*p* < 0.05; Student’s *t*-test) and reduced the expression of others related to the glycolytic pathway (*g6pdh, aldoA, eno1, hk1, ldha, pklr*) (Figure 1D, *n* = 8 ^∗∗^*p* < 0.001; Student’s *t*-test).

### CX3CL1 Induces Shape Changes of Microglia in Culture

**FIGURE 2:**
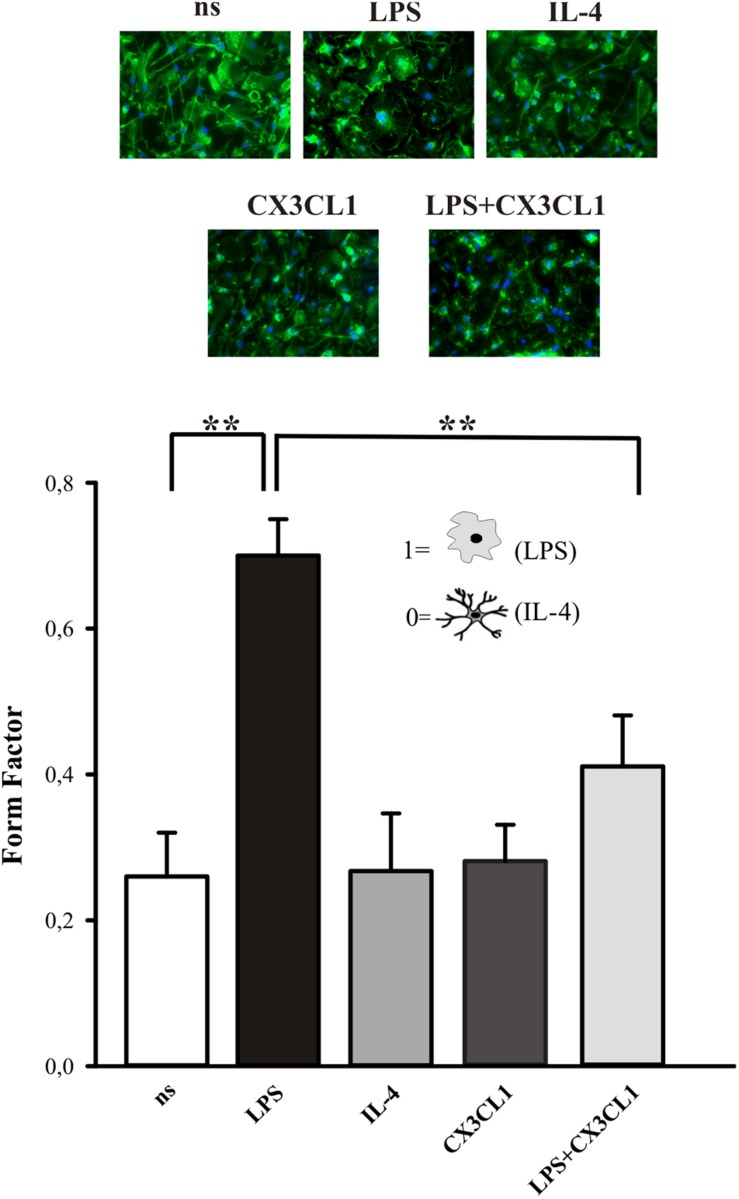
CX3CL1 induces change in microglia shape in culture. **Upper panel:** fluorescence images of microglia in culture stimulated with LPS, IL-4, CX3CL1, LPS + CX3CL1 and stained with Alexa-Fluor 488 Phalloidin. **Lower panel:** form factor analysis of microglia cells treated with LPS (pro-inflammatory stimulus) IL-4 (anti-inflammatory stimulus), CX3CL1, LPS + CX3CL1. Statistical analysis: Data are expressed as the mean (± SEM.) of *n* = 20 cells for each condition, ^∗∗^*p* < 0.001, Student’s *t-*test.

The activation state of microglia changes their shape, even if it is not possible to uniquely correlate a specific morphology to a specific phenotype (Kettenmann et al., 2011). We investigated the morphological changes of microglia in culture, upon CX3CL1 stimulation, calculating the “form factor” of single cells, that was calculated as described in the Method section. The form factor is 1 for round cells and correspondingly < 1 when the morphology deviates from the spherical shape. Microglia in culture displayed ramified processes with a small cell body and several long processes (Figure 2, not stimulated condition, ns). The results in Figure 2 show that microglia stimulated with LPS (100 ng/ml, 24 h), significantly change their form factor from 0.26 ± 0.06 (ns) to 0.7 ± 0.05; upon interleukine-4, (IL-4) treatment (20 ng/ml, 24 h) this parameter is similar to not stimulated cells (0.27 ± 0.08) in analogy with what previously reported (Grimaldi et al., 2016). The form factor of cells stimulated with CX3CL1 (0.28 ± 0.05) was similar to untreated cells and CX3CL1/LPS co-treatment significantly reverts the effect of LPS on cell shape variations (form factor: 0.41 ± 0.07) confirming that CX3CL1 efficiently contrasts the effects of inflammatory stimuli on microglial cells (*n* = 120 cells in total for each different conditions, ^∗∗^*p* < 0.001; one-way ANOVA followed by Mann–Whitney Rank Sum *post hoc* test).

### CX3CL1 Modulates Microglia Polarization in Pro-inflammatory Conditions *in vitro*

**FIGURE 3:**
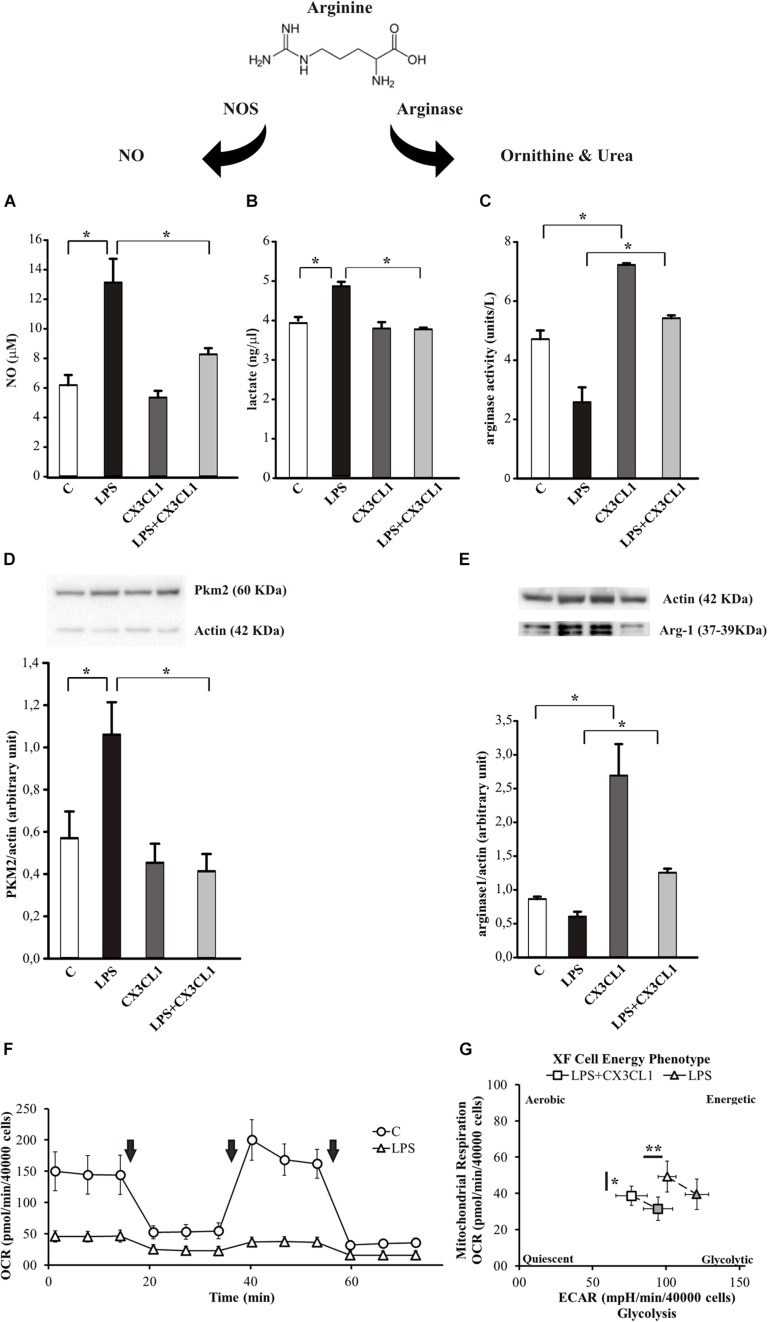
CX3CL1 modulates microglia phenotype in the context of pro-inflammatory conditions *in vitro*. **(A)** Release of NO by microglia cells not stimulated (C) or stimulated with LPS or CX3CL1 or LPS + CX3CL1; data are expressed as NO concentration (μM). **(B)** Lactate production by microglia cells not stimulated (C) or stimulated with LPS or CX3CL1 or LPS + CX3CL1; data are expressed as lactate concentration (ng/μl). **(C)** Arginase activity in microglia cells not stimulated (C) or stimulated with LPS or CX3CL1 or LPS + CX3CL1; data are expresses as units for liter. **(D)** Western-blot analysis of PKM2 protein expression in microglia cells incubated with vehicle (C) LPS, CX3CL1 or LPS + CX3CL1. At the top, representative image; at the bottom, histogram bar of the quantification of PKM2 expression (data are expressed as PKM2 signal normalized to Actin signal). **(E)** Western-blot analysis of Arg-1 protein expression in microglia cells incubated with vehicle (C) LPS, CX3CL1 or LPS + CX3CL1. At the top, representative image; at the bottom histogram bar of the quantification of Arg1 expression (data are expressed as Arg1 signal normalized to Actin signal). **(F)** Bioenergetic profile of microglial cells: the mitochondrial respiration of microglia, obtained by means of OCR by seahorse experiments, ± 10 ng/ml of LPS (48 h of treatment; triangle and circle, respectively). Arrows indicates the addiction of drugs used to specifically target the mitochondrial function (i.e., oligomycin, FCCP, rotenone + antimycin, in order of addiction). In principle, the first and the third drug lead to a dramatic OCR drop, while FCCP, as uncoupler agent, leads to a maximal oxygen consumption. While this behavior is observed in the untreated sample, LPS treatment abolished this kind of response. In the figure a representative experiment; values reported in the plot are the means of at least 8 replicates ± SD. **(G)** Energetic profile of LPS-treated microglia ± CX3CL1 treatment: the phenotype plot, where both ECAR and OCR are reported on the X and Y-axis, respectively, indicates that the LPS sample (10 ng/ml, open triangle) is more glycolytic than the LPS + CX3CL1-treated one (10 ng/ml + 100 nM, open square). This trend is maintained also under stressed conditions (gray symbols), where FCCP is not able to trigger a maximal respiratory capacity, as expected. In the figure a representative experiment is shown; values reported in the plot are the means of at least 8 replicates ± SD. Statistical analysis: Data are expressed as the mean (± SEM.) **(A)** *n* = 5, ^∗∗^*p* < 0.001 ^∗^*p* < 0.05, one-way ANOVA followed by Holm–Sidak *post hoc* test; **(C)** *n* = 6, ^∗^*p* < 0.05, one-way ANOVA followed by Tukey *post hoc* test; **(E)** *n* = 4,^∗^*p* < 0.05, one-way ANOVA followed by Tukey *post hoc* test. **(G)** *n* = 2, ^∗∗^*p* < 0.001 ^∗^*p* < 0.05, Student’s *t-*test.

Arginine metabolism via nitric oxide synthase (NOS) or arginase is at the crossroad of the pro- and anti-inflammatory microglia phenotypes (Orihuela et al., 2016). To verify the hypothesis that CX3CL1 could modulate microglia phenotype also under pro-inflammatory conditions, we stimulated cells with LPS (100 ng/ml, 24 h) and then with CX3CL1 (100 nM) for additional 24 h, and measured the release of nitric oxide (NO), the production of lactate and the arginase activity in microglia 24 h later. As shown in Figure 3A, LPS-induced NO release was significantly reduced by treatment with CX3CL1, bringing it back to the control level (*n* = 5, ^∗^*p* < 0.05; One- way ANOVA followed by Holm–Sidak *post hoc* test). In the same conditions we measured the lactate concentration in the medium of cultured microglia: Figure 3B shows that the LPS stimulation induced an increase in the lactate production that is reduced after CX3CL1 treatment (*n* = 3, ^∗^*p* < 0.05; One- way ANOVA followed by Tukey *post hoc* test). We also decided to analyze the expression of the Pyruvate Kinase isozymes M2 (PKM2) which is an enzyme involved in glycolysis that converts phosphoenolpyruvate to pyruvate and generates ATP. Data obtained by western blot assays reported in Figure 3D showed that PKM2 protein levels in microglia markedly increased after LPS exposure while they returned to the control level after CX3CL1 treatment (*n* = 3, ^∗^*p* < 0.05; One- way ANOVA followed by Holm–Sidak *post hoc* test). Regarding the arginase activity and arginase 1 (Arg-1) protein expression in microglia, cells were treated as described above and, as shown in Figure 3C, CX3CL1 increased arginase activity and reverted the reduction induced by LPS treatment (*n* = 6, ^∗^*p* < 0.05; One-way ANOVA followed by Tukey *post hoc* test). Moreover data reported in Figure 3E demonstrated that the stimulation with CX3CL1 (100 nM, 24 h) induced an increase in Arg-1 protein in microglia *vs.* not treated cells (*n* = 4, ^∗^*p* < 0.05, one-way ANOVA followed by Tukey *post hoc* test) and that the low expression level observed after LPS treatment is back at control level upon CX3CL1 stimulation. To further analyze the effect of fractalkine on the central metabolism, the energetic profile of microglia was assessed through the Cell Mito Stress Test (Agilent) using the extracellular flux analyzer XFe96 (Seahorse Bioscience, Houston, TX, United States). Briefly, this platform allows the determination of the Oxygen Consumption Rate (OCR) under both basal and stressed conditions which is indicative of the mitochondrial function; at the same time the extracellular acidification rate (ECAR) was also measured as an indirect quantification of the glycolytic process. The drugs used to promote the stressed condition target (according to the addition order) (i) complex V, i.e., ATP production; (ii) membrane potential, i.e., by uncoupling electron transfer and proton translocation: (iii) electron transfer chain, i.e., complex I and III. As expected (Voloboueva et al., 2013; Orihuela et al., 2016), LPS treatment (10 ng/ml, 48 h) promoted a deep metabolic re-programing as compared to the untreated sample: the OCR was dramatically reduced (∼65% lower; Figure 3F), while ECAR (and therefore glycolysis) was doubled (see Supplementary Figure S1A), shifting the aerobic profile of microglia to a glycolytic phenotype upon LPS treatment (see Supplementary Figure S1B). In light of this, we investigated whether CX3CL1 is able to attenuate the effect of LPS on the metabolic profile of microglia. Both untreated and LPS-treated samples were incubated with 100 nM CX3CL1 24 h after LPS treatment, and Seahorse analysis was then performed. While CX3CL1 did not affect the energetic profile of the untreated sample (see Supplementary Figure S2), in the LPS sample this chemokine promoted a significantly decrease of glycolysis and of OCR even though for the latter to a lower extent (Figure 3G and Supplementary Figure S3). The same trend was observed also at 100 ng/ml of LPS (data not shown).

### CX3CL1 Attenuates Microglial Activation After Ischemia *in vivo*

**FIGURE 4:**
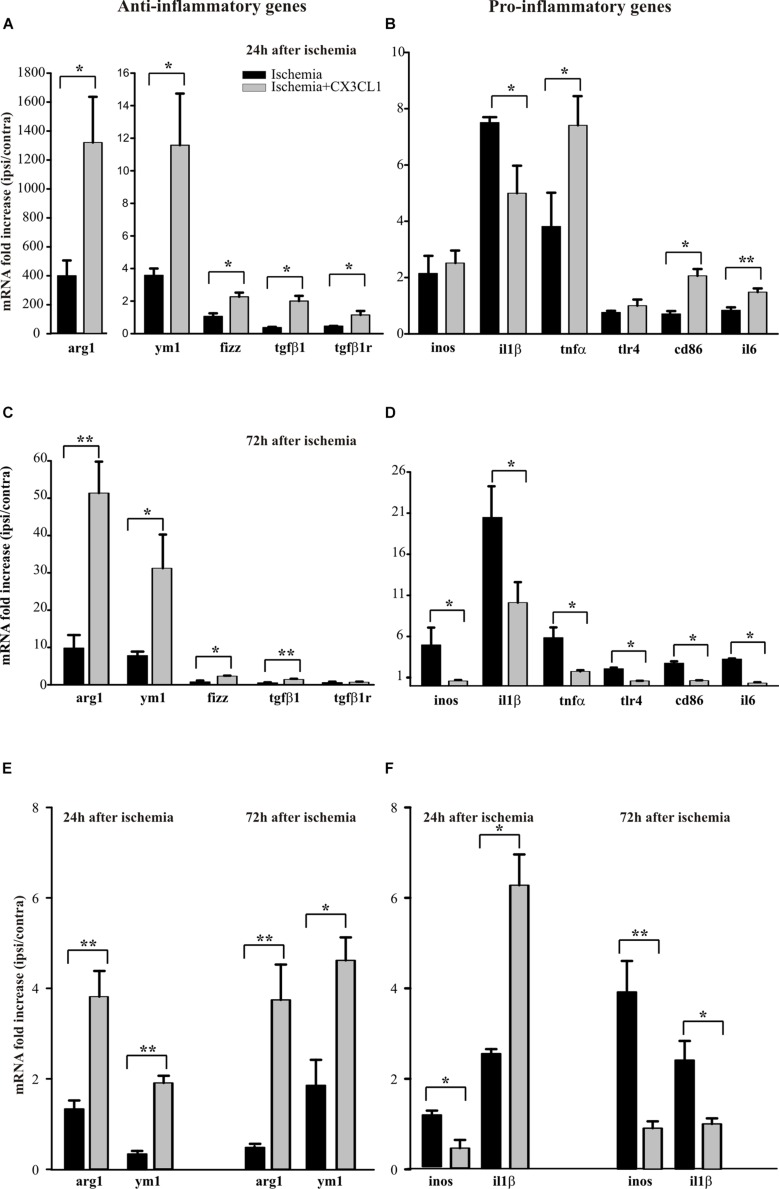
Effects of CX3CL1 in modulating microglia polarization state after ischemia. Expression analysis by RT-qPCR for mRNAs of anti-inflammatory (a*rg1, ym1*, *fizz, tgfβ1, tgfβ1r*), and pro-inflammatory (i*nos, il1β, tnfα, tlr4, cd86, il6*) related genes in Cd11b^+^ cells extracted from contra- and ipsi-lateral brain hemispheres of mice subjected to pMCAO 24 h **(A,B)** and 72 h **(C,D)** after the insult. Expression analysis by RT-qPCR for mRNAs of anti-inflammatory (a*rg1* and *ym1;* **(E)** and pro-inflammatory (i*nos* and *il1β*; **F)** related genes in microglial cells sorted by FACS from contra- and ipsi-lateral brain hemisphere cell suspensions of mice subjected to pMCAO 24 h and 72 h after the insult **(E,F)**. For each gene data are expressed as specific mRNA fold increase in the ipsilateral hemisphere of vehicle (black bars) or CX3CL1 treated (gray bars) mice normalized to the mRNA expression for the respective contralateral brain hemisphere. Statistical analysis: Data are expressed as the mean (± SEM.) of *n* = 6 **(A–D)** and *n* = 4 **(E,F)** mice per group, ^∗∗^*p* < 0.001, ^∗^*p* < 0.05, Student’s *t-*test.

We have previously demonstrated that exogenous CX3CL1 has a long-lasting neuroprotective action *in vivo* against pMCAO in rodents reducing neurological deficits and infarct size related with cerebral ischemia (Cipriani et al., 2011). We have now decided to investigate how CX3CL1 affects microglia phenotype during ischemia: to this aim mice were subjected to pMCAO, we verified that CX3CL1-treated mice had a reduced ischemic volume as previously shown (Cipriani et al., 2011), then CD11b^+^ cells were isolated from the ipsi- and contra-lateral brain hemispheres 24 or 72 h after ischemia and gene expression was analyzed by RT-qPCR. As reported in Figure 4A, at 24 h after ischemia CD11b^+^ cells increased their expression of anti-inflammatory genes (*arg1*, *ym1*, *fizz*, *tgfβ1*, *tgfβ1r*) in the ipsilateral hemisphere, upon CX3CL1 treatment (*n* = 6 mice per group, ^∗^*p* > 0.05; Student’s *t*-test).

Instead the expression pattern of the pro-inflammatory genes analyzed (*inos, il1β, tnfα, tlr4, cd86, il6*) is less clear: in particular, we observed an increase in the expression of *tnfα*, *cd86* e *tlr4* in CX3CL1 treated mice, while CX3CL1 reduced *il1β* expression (*n* = 6 mice per group, ^∗∗^*p* < 0.001 ^∗^*p* > 0.05; Student’s *t*-test). Since it was demonstrated that microglia phenotype changes from anti- to pro-inflammatory with the progression of cerebral ischemia (Fumagalli et al., 2015; Ma et al., 2017) we isolated CD11b^+^ cells from the ipsi- and contra-lateral brain hemispheres of mice at 72 h after ischemia and analyzed by RT-qPCR the same set of genes.

Data reported in Figure 4C show that CD11b^+^ cells isolated from the ipsilateral hemisphere 72 h after ischemia induction, express higher level of pro-inflammatory genes that undergo a significant reduction of expression upon CX3CL1 treatment (*n* = 6 mice per group, ^∗^*p* > 0.05; Student’s *t*-test). Interestingly, most of the anti-inflammatory genes considered (Figure 4C) are still increased in the ipsilateral hemisphere of CX3CL1 treated mice (*n* = 6 mice per group, ^∗∗^*p* < 0.001 ^∗^*p* > 0.05; Student’s *t*-test). The MACS column system does not permit to distinguish microglia from other myeloid resident cells and from infiltrating immune cells during the ischemic insult, because they can express CD11b as well as CX3CR1. For this reason, we used a new animal cohort and performed gene analysis only on microglia after pMCAO, taking advantage of the fluorescence-activated cell sorting. Starting from CD45 positive low population, microglial cells were isolated as CD11b positive Ly6C/Ly6G double negative cells from both the ipsi- and contra-lateral brain hemispheres, 24 or 72 h after ischemia and gene expression was analyzed by qRT-PCR. With this gating strategy we uncovered a microglia fraction of 80–90% among CD45^+^ cells at all time points and an immune cell infiltrate (CD45^high^ among CD45^+^ cells) of 16.37 ± 6.63% saline- *versus* 19.42 ± 5.77% CX3CL1-treated mice at 24 h after ischemia and of 10.87 ± 1.87% saline- *versus* 20.07 ± 2.71% CX3CL1-treated mice at 72 h after ischemia, confirming the chemotactic role of CX3CL1. FACS analysis allowed us to recover an average of 20 × 10^3^ microglial cells per hemisphere, that is not enough to analyze the expression of all the genes taken into consideration in the previous panels; we have therefore chosen only a few of them. Data reported in Figures 4E,F confirmed that CX3CL1 treatment increased the expression of the anti-inflammatory genes *arg1* and *ym1* and decreased that of pro-inflammatory gene *inos* on microglial cells isolated from brain hemispheres both 24 and 72 h after ischemia. Regarding the CX3CL1 effect on *il1β* expression in microglial cells after brain ischemia, it induced an increase in the expression of this cytokine 24 h after ischemia while it had an opposite effect after 72 h from ischemic damage (*n* = 4 mice per group, ^∗∗^*p* < 0.001 ^∗^*p* > 0.05; Student’s *t*-test), contrary to what observed for CD11B^+^ cells (see Figures 4B,D).

### CX3CL1 Modulates Microglial Metabolic State After Ischemia *in vivo*

Several evidence suggest a role of metabolic reprograming in the regulation of the innate inflammatory response of microglia (Voloboueva et al., 2013; Gimeno-Bayón et al., 2014). We analyzed the expression profiles of genes related to the glycolytic and oxidative pathways in CD11b^+^ cells extracted from the ipsi- and contra-lateral brain hemispheres of mice treated with CX3CL1, at 24 and 72 h after ischemia induction.

**FIGURE 5:**
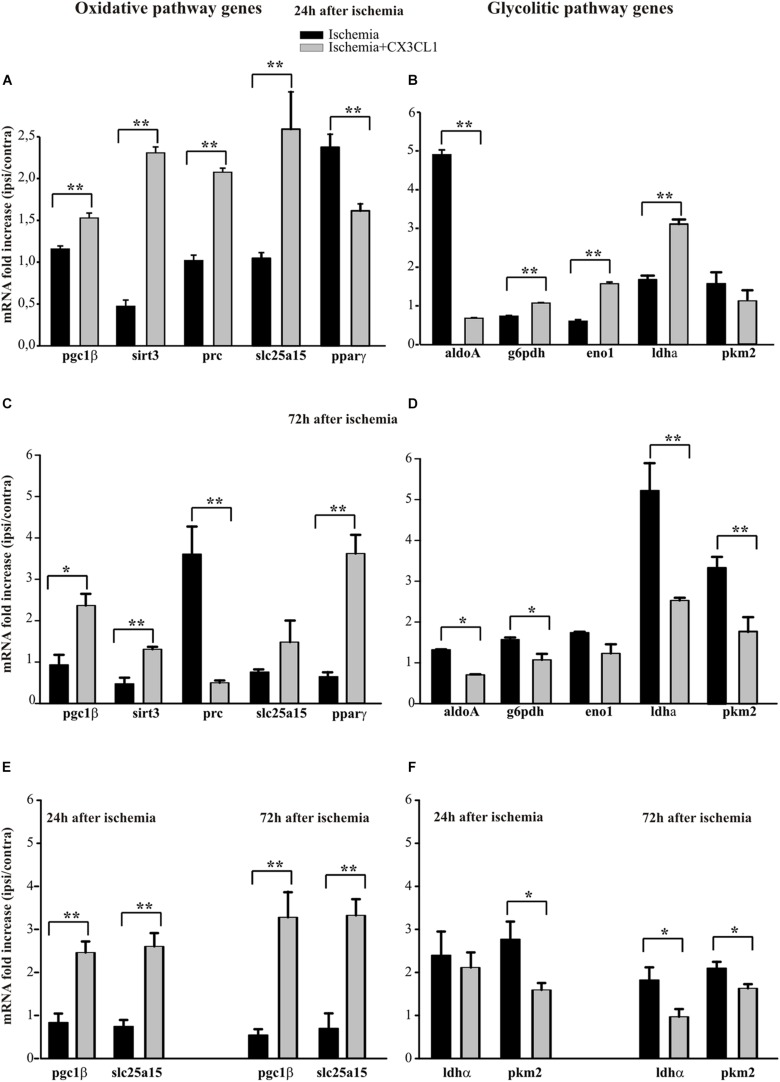
Effects of CX3CL1 in modulating microglia metabolic state after ischemia. Expression analysis by RT-qPCR for mRNAs of oxidative pathway (*pgc1*β, *sirt3*, *prc*, *slc25a15 ppar*γ) and glycolytic pathway (*aldoA, g6pdh, eno1, ldha, pkm2*) related genes in Cd11b^+^ cells extracted from contra- and ipsi-lateral brain hemispheres of mice subjected to pMCAO 24 h **(A,B)** and 72 h **(C,D)** after the insult. Expression analysis by RT-qPCR for mRNAs of oxidative (*pgc1β* and *slc25a15*; **(E)** and glycolytic pathway (*ldha* and *pkm2*; **(F)** related genes in microglial cells sorted by FACS from contra-and ipsi-lateral brain hemisphere cell suspensions of mice subjected to pMCAO 24 h and 72 h after the insult **(E,F)**. For each gene data are expressed as specific mRNA fold increase in the ipsilateral hemisphere of vehicle (black bars) or CX3CL1 treated (gray bars) mice normalized to the mRNA expression for the respective contralateral brain hemisphere. Statistical analysis: Data are expressed as the mean (± SEM.) of *n* = 6 **(A–D)** and *n* = 4 **(E,F)** mice per group, ^∗∗^*p* < 0.001, ^∗^*p* < 0.05, Student’s *t-*test.

The analysis of some genes involved in the oxidative pathway (such as *pgc1*β, *sirt3*, *prc*, *slc25a15* and *ppar*γ) shows that CX3CL1 increases their expression in the ipsilateral region at 24 h and 72 h after ischemia with the exception of *pparγ*, whose expression is reduced at 24 h (Figure 5A, *n* = 6 mice per group, ^∗∗^*p* < 0.001; Student’s *t*-test) and of *prc*, that decreased at 72 h (Figure 5C, *n* = 6 mice per group, ^∗∗^*p* < 0.001 ^∗^ *p* > 0.05; Student’s *t*-test). On the other hand, among the genes associated with the glycolytic pathway (such as *aldoA, g6pdh, eno1*, and *ldha*), (Figure 5B) at 24 h only *aldoA* was reduced (*n* = 6 mice per group, ^∗∗^*p* < 0.001; Student’s *t*-test). In contrast 72 h after ischemia they were all reduced upon CX3CL1 treatment (Figure 5D; *n* = 6 mice per group, ^∗∗^*p* < 0.001 ^∗^*p* > 0.05; Student’s *t*-test). We performed a similar analysis on microglial cells isolated from brain hemispheres by fluorescence-activated cell sorting 24 and 72 h after ischemia and what we observed is an increase in the expression of *pgc1β* and *slc25a15* in CX3CL1 treated mice both 24 and 72 h after ischemia (Figure 5E, *n* = 4 mice per group, ^∗∗^*p* < 0.001 ^∗^*p* > 0.05; Student’s *t*-test), while CX3CL1 reduced *pkm2* expression 24 and 72 h after brain damage and ldhα expression 72 h after ischemia insult (Figure 5F, *n* = 4 mice per group, ^∗∗^*p* < 0.001 ^∗^*p* > 0.05; Student’s *t*-test).

## Discussion

### Microglia and Neuroinflammation

Neuroinflammation is associated with the pathophysiology of neurodegenerative disorders (Leszek et al., 2016), and glial activation during neuroinflammation is a common feature in disease progression, resulting in the production of inflammatory cytokines, such as tumor necrosis factor-α (TNF-α), interleukin-1β (IL-1β), reactive oxygen species, and nitric oxide, which can ultimately lead to neuronal loss (Meraz-Ríos et al., 2013). As CNS-resident immune cells, microglia play a key role in maintaining tissue homeostasis, as well as in inducing neurotoxicity (Liddelow et al., 2017). Under normal oxygen conditions, cells usually acquire energy via two mechanisms: glucose is converted to pyruvate via glycolysis which moves to mitochondria to produce ATP through oxidative phosphorylation (Dashty, 2013). Under hypoxic conditions, anaerobic glycolysis converts pyruvate into lactate (Murray, 2006; Hardie, 2007). Immune cells switch from oxidative phosphorylation to aerobic glycolysis, as described in other cell types (Warburg effect) (Warburg, 1956; Vander Heiden et al., 2009), in order to produce metabolic resources necessary to satisfy the request of cell proliferation and activation. This metabolic reprograming plays a key role in the process of the innate inflammatory response; in particular, the pro-inflammatory state is correlated with a shift of energy production from oxidative phosphorylation to aerobic glycolysis (Krawczyk et al., 2010; O’Neill and Hardie, 2013; Orihuela et al., 2016). Moreover, glycolysis inhibition reduces the pro-inflammatory polarization of immune cells, indicating a possible regulatory role in inflammatory cell function (Cheng et al., 2014). When stimulated with LPS/IFN-γ, microglia switch toward a pro-inflammatory phenotype, undergo aerobic glycolysis and release pro-inflammatory cytokines; when stimulated with IL-4/IL-13, they acquire an anti-inflammatory phenotype, with inflammation resolution and tissue repair (Kelly and O’Neill, 2015). In this view, energy demand would be associated with specific functional activities such as change in morphology, phagocytosis and translocation to the injured site of microglial cells (Stence et al., 2001; Koizumi et al., 2007; Fu et al., 2014), and thus may influence the contribution of microglia activation to various neurodegenerative condition. There are evidence of both harmful and protective roles of microglia in stroke (Nakajima and Kohsaka, 2004; Neumann et al., 2006; Neher et al., 2013), and so the precise role and timing of microglia in stroke and cerebral ischemia is debated. Activated microglia produce pro-inflammatory cytokines, nitric oxide and reactive oxygen species inducing an acute and chronic inflammatory response which exacerbates neuronal injury in stroke (Block et al., 2007). Nevertheless, both exogenous and resident proliferating microglia are able to exert neuroprotective action in brain ischemia, through the production of neurotrophic factors (Imai et al., 2007; Lalancette-Hébert et al., 2007; Narantuya et al., 2010). This dual activity of microglia might be explained by the differences in the parenchymal milieu composition produced in injuries of diverse entity and/or at distinct stages of the pathology, which might have various effects on microglia activation (Lai and Todd, 2008). In this contest, the molecular mechanisms through which microglia activate different responses have become an important area of ischemia research. CX3CL1 has been reported to inhibit the release of inflammatory cytokines (Zujovic et al., 2000; Mizuno et al., 2003; Cardona et al., 2006) and reduce microglia activation, keeping these cells in what (before the deep use of transcriptome analysis) has been defined an “off” state (Biber et al., 2007). We previously demonstrated that CX3CL1 is protective against cerebral ischemia (Cipriani et al., 2011). Since it was demonstrated that during the progression of cerebral ischemia microglia phenotype changes from anti- to pro-inflammatory (Fumagalli et al., 2015; Ma et al., 2017) and microglia express CX3CR1, we investigated whether the neuroprotective effect of CX3CL1 in ischemia is due to its ability to change the phenotype of microglia. For this reason, we analyzed the expression profiles of anti- and pro-inflammatory genes and those related to the metabolic reprograming, in the regulation of the inflammatory response both *in vitro* and *in vivo*.

### CX3CL1 Modulates the Inflammatory Phenotype of Microglia *in vitro*

In this paper we demonstrate that CX3CL1 drives microglia toward an anti-inflammatory phenotype reducing the expression of pro-inflammatory genes and increasing the expression of those related to an anti-inflammatory state. Moreover, CX3CL1 switched the metabolic state of microglia from glycolysis to oxidative pathway, increasing the expression of gene related to oxidative phosphorylation and decreasing those involved in glycolytic metabolism of glucose. These effects are in line with what was observed *in vitro* with NO, lactate and arginase activity in primary microglia in the context of inflammatory environment (induced by LPS stimulation), where CX3CL1 counteracts the acquisition of a pro-inflammatory phenotype. To provide specific experimental data on the bioenergetics profile of microglia following pro-inflammatory activation and CX3CL1 treatment, primary microglia cells were seeded in a Seahorse XF plate in order to detect the cellular oxygen consumption rate (OCR) and extracellular acidification rate (ECAR). First, we observed a normal response pattern for unstimulated primary microglia and a decrease in basal respiration in LPS-treated cells that were unresponsive to the mitochondrial stressors, suggesting an impairment of mitochondrial function. More interesting, while microglia exposed to CX3CL1 maintained a bioenergetics profile similar to control condition, CX3CL1 promoted a significant decrease of glycolysis and OCR in LPS-stimulated microglia, confirming that the different phenotypes of microglia induced by CX3CL1 treatment are associated with different metabolic programing.

### CX3CL1 Modulates the Inflammatory Phenotype of Microglia *in vivo*

It is known that injured neurons respond to toxic insults by releasing CX3CL1, which is upregulated, cleaved, and released upon ischemia or excitotoxic insult (Chapman et al., 2000; Tarozzo et al., 2002; Limatola et al., 2005; Noda et al., 2011). The neuroprotective or neurotoxic action of CX3CL1 likely depends on the activation (priming) state of microglia in the different phases of acute and chronic brain diseases (Dénes et al., 2008; Cho et al., 2011; Cipriani et al., 2011; Liu et al., 2011; Pabon et al., 2011; Shan et al., 2011; Fumagalli et al., 2013; Lauro et al., 2015). In this view, we hypothesized that CX3CL1 is neuroprotective, in permanent focal cerebral ischemia, due to its ability to modulate microglia polarization and activation state toward a protective one. To confirm its role in modulating microglia polarization also during ischemia *in vivo*, we isolated CD11b^+^ cells from the ipsi- and contra-lateral cerebral hemispheres of mice subjected to pMCAO, at 24 and 72 h after ischemic insult, and analyzed the expression of the same genes taken in consideration in the *in vitro* experiments. It was demonstrated that upon acute brain damages, microglia cells produce anti-inflammatory cytokines and trophic factors at the site of injury to promote restorative processes (Neumann et al., 2006; Perego et al., 2011; Hu et al., 2012; Ma et al., 2017). However, over time, microglia acquire a pro-inflammatory phenotype releasing pro-inflammatory cytokines, chemokines, and inducible nitric oxide synthase, which results in the exacerbation of brain damage (Shi and Pamer, 2011). We observed that CX3CL1 changes the phenotype of CD11b+ cells isolated in the pMCAO mice model and that this effect is time dependent, according with the appearance of anti-inflammatory (at 24 h) and pro-inflammatory microglia (at 72 h) (Schroeter et al., 1997; Hu et al., 2012; Fumagalli et al., 2015). In fact, CX3CL1 induced an increase in the expression of several anti-inflammatory and oxidative pathway-related genes already 24 h after ischemia and this effect remained up to 72 h. On the contrary, it down-modulated the expression of several pro-inflammatory and glycolytic pathway markers only at 72 h after the ischemic insult, suggesting that in a way CX3CL1 acts potentiating the function of the anti-inflammatory microglia, trying to prolong this population phenotype. Since CD11b^+^ cells contain a mixture of myeloid cells, we specifically sorted microglial cells (24 or 72 h after ischemia) from both the ipsi- and contra-lateral brain hemisphere by flow cytometry to specifically analyze their gene expression. With this gating strategy we uncovered a microglia fraction of 80–90% among CD45^+^ cells and we repeated on these cells the expression analysis for some of the genes evaluated on CD11b^+^ cells. In this way we confirmed that CX3CL1 induces an increase in the expression of anti-inflammatory and oxidative pathway related genes and decreases that of pro-inflammatory and glycolytic pathway markers on microglia. Since in our experimental condition the immune cell infiltrate (CD45^high^ among CD45^+^ cells) in CX3CL1-treated mice is about 20%, both at 24 and 72 h after ischemia, we cannot ruled out that what we observed for the CD11b^+^ cells included the contribution of the infiltrating immune cells. Furthermore, it is possible that CX3CL1 modulates the activation state of these cells too in order to recover the ischemic damage. For example, this would explain the observed differences regarding the CX3CL1 effect on il1β expression. It would be interesting to analyze the infiltrating population and the activation state of these cells in the presence and in the absence of the CX3CL1 stimulus and this will probably be the subject of a subsequent study. Moreover, we interpret that CX3CL1 does not modulate all the markers taken in consideration, because the injury-induced inflammatory processes are dynamic, with spatial and temporal heterogeneity, and/or because some microglial cells have intermediate phenotypes, with the simultaneous expression of pro- and anti-inflammatory genes (Ito et al., 2001; Taylor and Sansing, 2013; Morrison and Filosa, 2013). More interestingly, the ability of CX3CL1 to induce a metabolic switch toward oxidative metabolism in microglia might be necessary to accommodate the beneficial role of anti-inflammatory microglia and results in the generation of metabolites, which are harmful for neuronal survival. Indeed, CX3CL1 can act in concert with other chemokines, growth factors and metabolites to counteract glutamate excitotoxic damage with a mechanism that involves neurons, microglia and astrocytes (Lauro et al., 2010; Rosito et al., 2014). Considering all these data, we hypothesize that, in addition to limit neuronal damage counteracting excitotoxicity, the release of CX3CL1 in response to ischemic insult might be a physiological response of brain tissue to trigger neuroprotection, modulating the activation state of microglia and its metabolism in order to limit neuro-inflammation and give more time to the cells of the brain parenchyma to organize a neuroprotective response. In this view CX3CL1 may be an important messenger molecule that plays a part in microglia response to extracellular signals during ischemic injuries, linking anti-inflammatory microglia to reduced injury and potential repair and could represent a possible target to modulate microglia phenotype to limit inflammation upon ischemia.

## Ethics Statement

The experiments described in the present work, were approved by the Italian Ministry of Health in accordance with the guidelines on the ethical use of animals from the European Community Council Directive of September 22, 2010 (2010/63/EU).

## Author Contributions

CLa and CLi contributed to the conception and design of the study. CLa designed and performed all the experiments and statistical analysis and wrote the manuscript. GC performed the *in vivo* experiments. LM performed the real time PCR analysis. FA and GP designed and performed isolation of microglia cells by cell sorting. AP and SR ran and calculated the seahorse experiments. FC supervised the Hyp-ACB facility and data analysis. All authors contributed to the manuscript revision, and read and approved the submitted version.

## Conflict of Interest Statement

The authors declare that the research was conducted in the absence of any commercial or financial relationships that could be construed as a potential conflict of interest.

## Supplementary Material

FIGURE S1 Bioenergetic profile of microglial cells. **(A)** The glycolysis of microglia ± 10 ng/ml of LPS as ECAR parameter of the seahorse experiment reported in Figure 3F, indicates that the LPS treatment doubled the basal value. **(B)** The effect of LPS on microglia is reported as phenotype plot. This plot, where both ECAR and OCR are reported on the *X* and *Y*-axis, respectively, indicates that the LPS sample (open triangle) is more glycolytic and less aerobic than the untreated one (open circle). Interestingly, while the untreated responds to stressors as expected (gray circles), by increasing both OCR and ECAR, the LPS sample is quite insensitive to drugs treatment (gray triangles). This could be ascribed to a poor and probably maximal basal mitochondrial activity. In the figure a representative experiment; values reported in the plot are the means of at least 8 replicates ± SD.

FIGURE S2 Bioenergetic profile of microglial cells. The mitochondrial respiration of microglia, obtained by means of OCR by seahorse experiments, ± 100 nM of CX3CL1 (24 h of treatment; black and empty symbols, respectively). Arrows indicate the addiction of drugs used to specifically target the mitochondrial function (i.e., oligomycin, FCCP, rotenone + antimycin, in order of addiction). Chemokine treatment does not affect the energetic profile. In the figure a representative experiment; values reported in the plot are the means of at least 8 replicates ± SD.

FIGURE S3 Energetic profile of LPS-treated microglia ± CX3CL1 treatment. LPS sample (10 ng/ml, open triangle) and LPS + CX3CL1-treated one (10 ng/ml + 100 nM, open square). Arrows indicates the addiction of drugs used to specifically target the mitochondrial function (i.e., oligomycin, FCCP, rotenone + antimycin, in order of addiction). In the figure a representative experiment is shown as OCR **(A)** and ECAR **(B)** measurements. Histograms from panels **(C–H)** (LPS sample, black square and LPS + CX3CL1-treated sample, open square) are belong to panels **(A,B)** data, according to Agilent Mito Stress Report generator. Values reported in the plot are the means of at least 8 replicates ± SD.
