# Supplementary figures and images for: Fractalkine Modulates Microglia Metabolism in Brain Ischemia
Source: Front Cell Neurosci. 2019 Sep 13;13:414. doi: 10.3389/fncel.2019.00414 (PMC6755341; doi:10.3389/fncel.2019.00414)

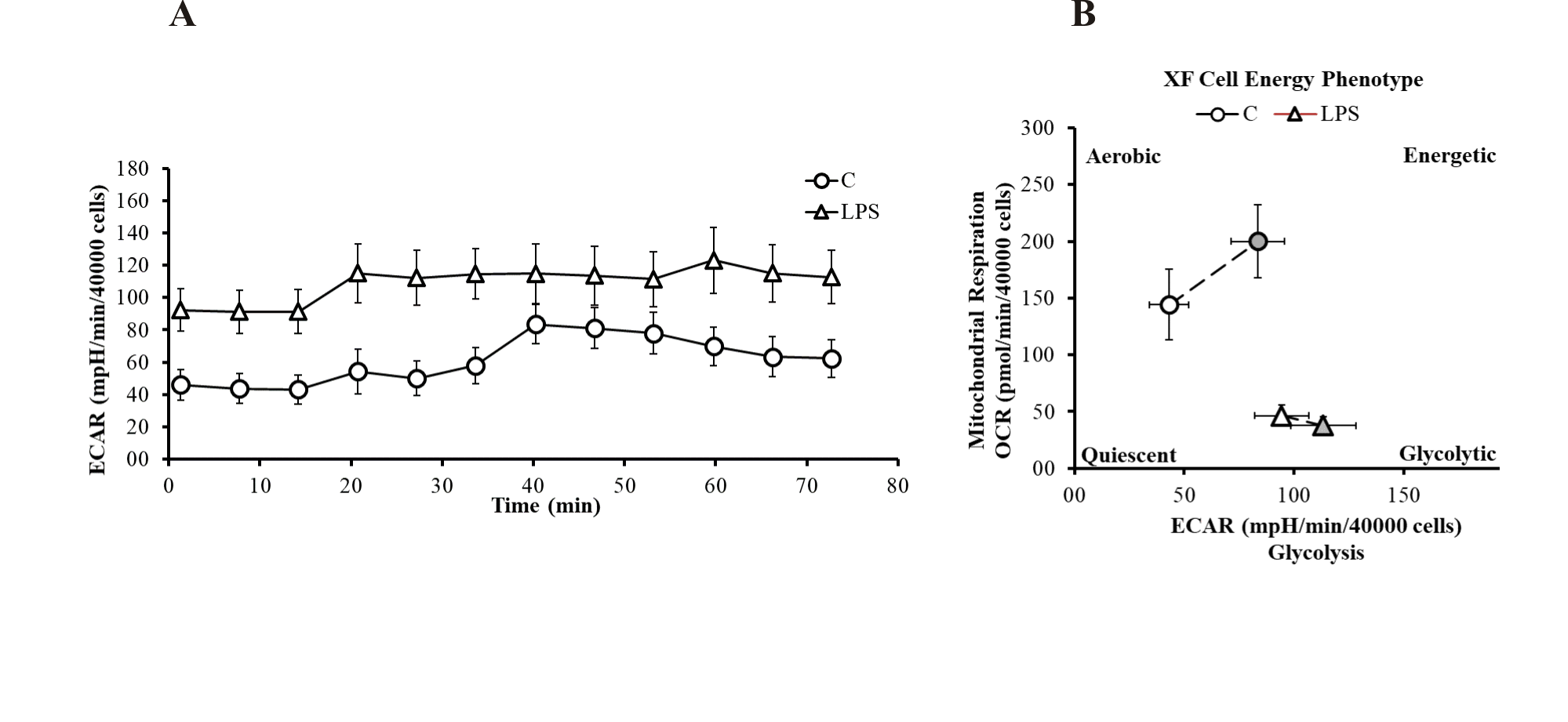

Supplement: FIGURE S1 — Bioenergetic profile of microglial cells. (A) The glycolysis of microglia ± 10 ng/ml of LPS as ECAR parameter of the seahorse experiment reported in Figure 3F, indicates that the LPS treatment doubled the basal value. (B) The effect of LPS on microglia is reported as phenotype plot. This plot, where both ECAR and OCR are reported on the X and Y-axis, respectively, indicates that the LPS sample (open triangle) is more glycolytic and less aerobic than the untreated one (open circle). Interestingly, while the untreated responds to stressors as expected (gray circles), by increasing both OCR and ECAR, the LPS sample is quite insensitive to drugs treatment (gray triangles). This could be ascribed to a poor and probably maximal basal mitochondrial activity. In the figure a representative experiment; values reported in the plot are the means of at least 8 replicates ± SD. [file Image_1.TIF]

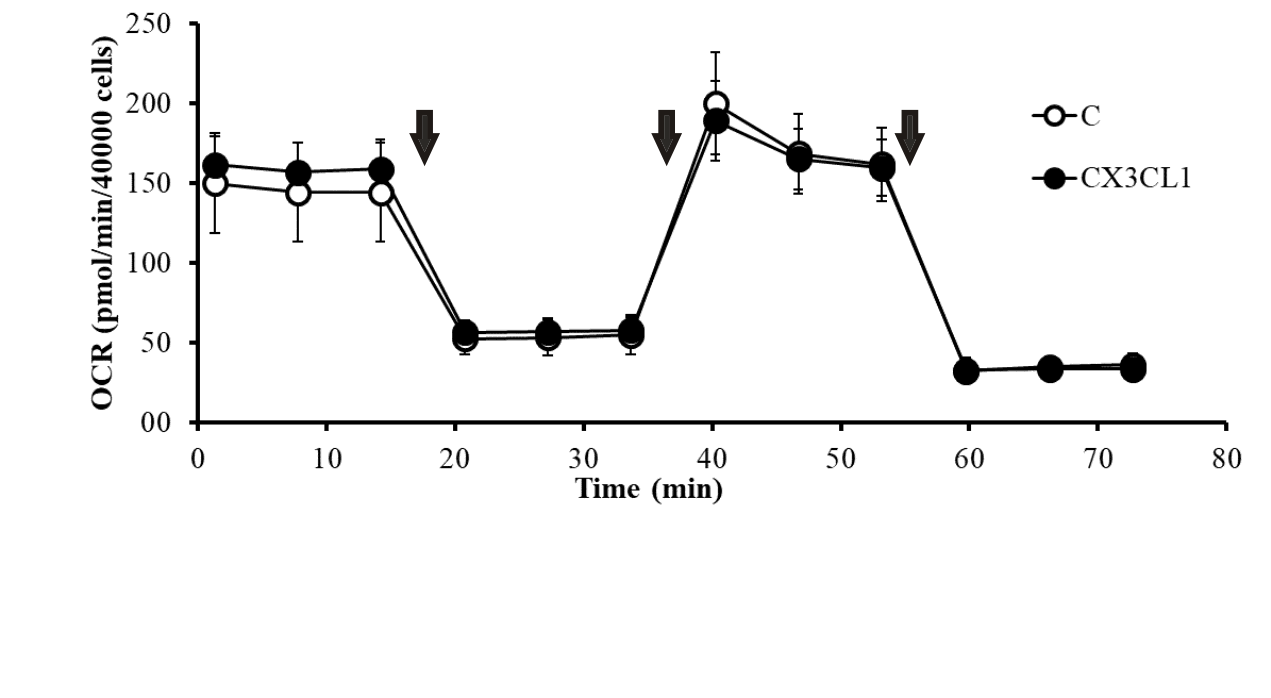

Supplement: FIGURE S2 — Bioenergetic profile of microglial cells. The mitochondrial respiration of microglia, obtained by means of OCR by seahorse experiments, ± 100 nM of CX3CL1 (24 h of treatment; black and empty symbols, respectively). Arrows indicate the addiction of drugs used to specifically target the mitochondrial function (i.e., oligomycin, FCCP, rotenone + antimycin, in order of addiction). Chemokine treatment does not affect the energetic profile. In the figure a representative experiment; values reported in the plot are the means of at least 8 replicates ± SD. [file Image_2.TIF]

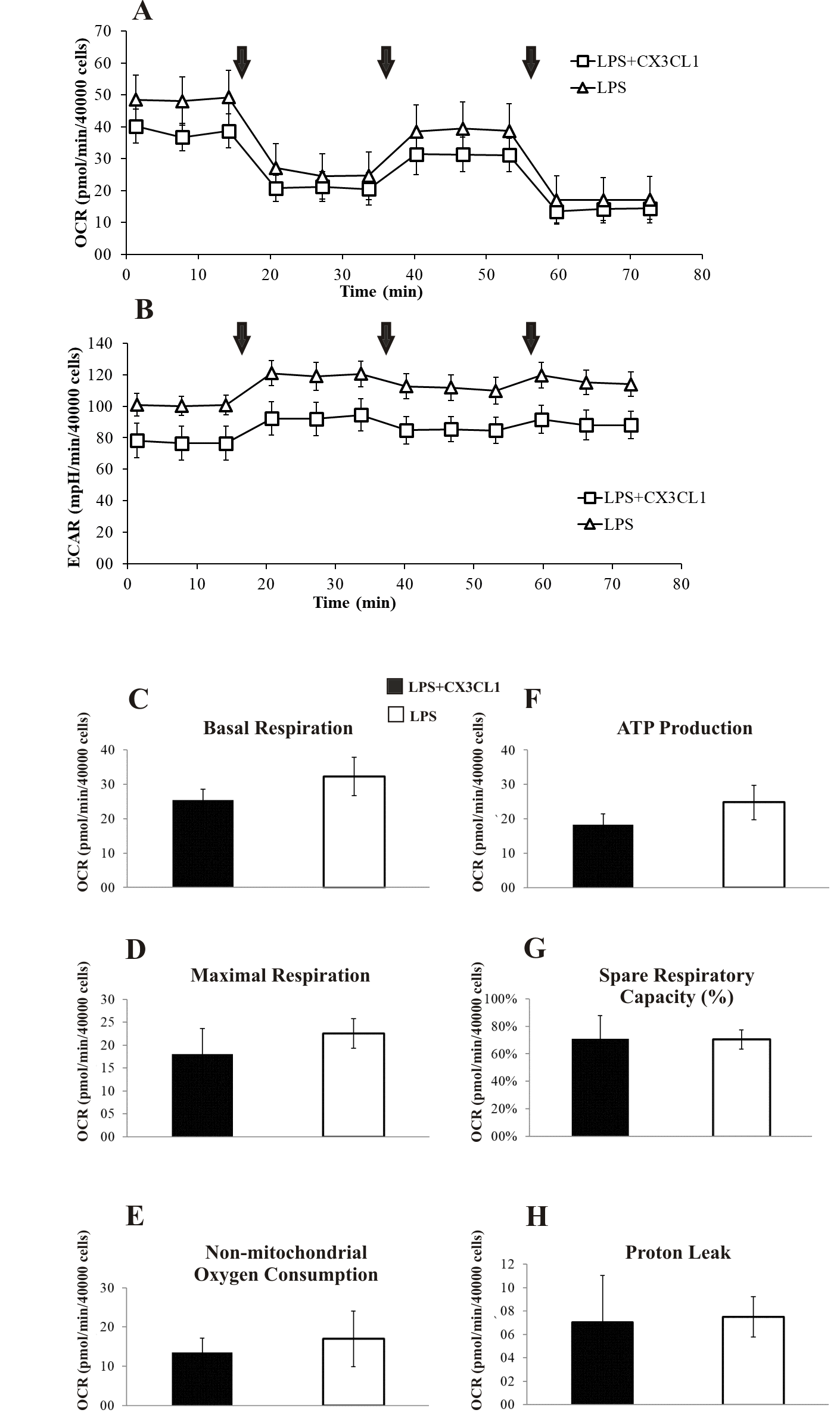

Supplement: FIGURE S3 — Energetic profile of LPS-treated microglia ± CX3CL1 treatment. LPS sample (10 ng/ml, open triangle) and LPS + CX3CL1-treated one (10 ng/ml + 100 nM, open square). Arrows indicates the addiction of drugs used to specifically target the mitochondrial function (i.e., oligomycin, FCCP, rotenone + antimycin, in order of addiction). In the figure a representative experiment is shown as OCR (A) and ECAR (B) measurements. Histograms from panels (C–H) (LPS sample, black square and LPS + CX3CL1-treated sample, open square) are belong to panels (A,B) data, according to Agilent Mito Stress Report generator. Values reported in the plot are the means of at least 8 replicates ± SD. [file Image_3.TIF]
